# Supplementary material for: A chimeric vector for dual use in cyanobacteria and Escherichia coli, tested with cystatin, a nonfluorescent reporter protein
Source: PeerJ. 2021 Nov 3;9:e12199. doi: 10.7717/peerj.12199 (PMC8571960; doi:10.7717/peerj.12199)
Supplement: Supplemental Information 1 [file peerj-09-12199-s001.docx]

**Supplemental Article S1**

**A chimeric vector for dual use in cyanobacteria and *Escherichia coli*, tested with cystatin, a nonfluorescent reporter protein**

Mojca Juteršek and Marko Dolinar

**The newly constructed pMJc01 exhibits structural instability in the chassis with decreased LacI activity**

We observed structural instability of pMJc01 when it was isolated from *E. coli* XL1-Blue and transferred to chemically competent *E. coli* DH5α cells. Although DH5α transformants grew normally, pMJc01 isolated from the overnight cultures was approximately 1500-2000 bp longer than expected and had additional *Pst*I and *Bam*HI recognition sites. Restriction analysis showed that the unexpected length was due to a region derived from RSFmob-I. When the parent RSFmob-I vector was examined, it also showed different behaviour in XL1-Blue compared to the DH5α strain, with unexpected restriction sites and fragment lengths only in DH5α. In addition, PCR with the RSFmob-I template isolated from DH5α cells and the RSFmobF and RSFmobR primers (Table 1) resulted in non-specific products. Since the *E. coli* strains tested differ in cellular LacI content (LacI is overexpressed in XL1-Blue but not in DH5α) and the pMJc01 replication proteins are under the expression control of P_lacUV5_, we tested whether LacI affects the stability of pMJc01. IPTG was added to the growth medium of *E. coli* XL1-Blue cells transformed with intact pMJc01. After overnight incubation, the isolated plasmid showed the same characteristics as pMJc01 isolated from DH5α cells (increased size and additional restriction sites, see Suppl. Fig. 1). This suggests that the observed pMJc01 instability most likely results from reduced cellular LacI activity, either due to lower expression of the repressor protein (as in DH5α) or its release from the operator region (as in XL1-Blue in the presence of the IPTG inducer).


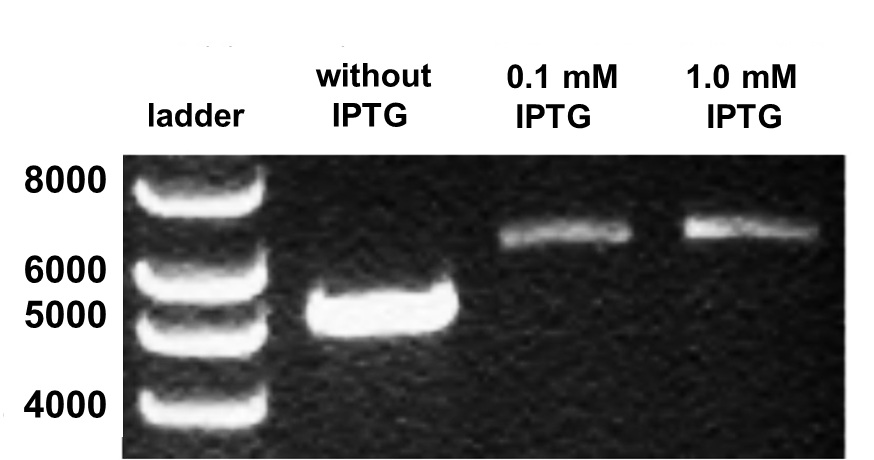


**Suppl. Art. Fig.1 Instability of pMJc01 in *E. coli* XL1-Blue grown in the presence of IPTG.** Agarose gel of pMJc01_EV isolated after overnight incubation in *E. coli* XL1-Blue in the absence or presence of either 0.1 or 1.0 mM IPTG. The expected size of pMJc01 EV is 5072 bp.

As mentioned in the main article, the P_lacUV5_ promoter can be exchanged for other promoters by utilizing *Bgl*II and *Bam*HI restriction sites upstream and downstream, shown in the schema below.

…CCCGCAGGGCCTGTCTCGGTCGATCATTCAGCCCGGCTCAT**AGATCT**GCGGGCAGTGAGCGCAACGCAATTAATGTAAGTTAGCTCACTCATTAGGCACCCCAGGCTTTACACTTTATGCTTCCGGCTCGTATAATGTGTGGAATTGTGAGCGGATAACAATTTCACACAGGATCTA**GGATCC**GGGGGGTGGCCCGATGAAGAACGACAGGACTTTGCAGGCCATA…

Magenta highlight marks the last 30 nucleotides of oriV sequences, yellow the P_lacUV5_ sequence and green the first 30 nucleotides of *repB* gene. *Bgl*II and *Bam*HI restriction sites are shown in bold red. The starting C of the shown sequence corresponds to position 403 in the nucleotide sequence of the plasmid deposited in GenBank (MN201591.1).

**Fluorescence measurements of cell cultures with different optical densities can lead to erroneous calculations of the average fluorescence per cell**

Because the relative fluorescence values for cultures of *E. coli* harbouring plasmids with the regulatory construct J23101_B0032 (1 : 0.5 : 1.7 - see main text for details) differed from other ratios (in particular the relative value for the pSB3K3 plasmid), we investigated whether this deviation was due to specific experimental conditions.

From the same overnight cell culture (*E. coli* XL1-Blue transformed with pMJc01_EV or pMJc01_RBS*_L21_GFPmut3b), dilution series were prepared with triplicates at different Abs_600_ values and fluorescence was measured (Suppl. Fig. 2). We observed a linear increase in fluorescence intensity for cells with expressed GFPmut3b (Suppl. Fig. 2a) and cells with empty pMJc01 plasmid (Suppl. Fig. 2b). However, when fluorescence was divided by optical density to represent average fluorescence per cell, which is a common practice in experiments where GFP is used as a reporter in biological parts measurements, values were significantly higher at lower optical densities and decreased exponentially with increasing Abs_600_ (Suppl. Fig. 2, black squares). This is likely a consequence of the scattering of incident and/or emitted light in turbid cell culture samples, reducing the relative amount of emitted light reaching the detector.

To apply this observation to the deviation of fluorescence ratio in cultures expressing GFPmut3b under control of the regulatory sequence J23101_B0032, we checked the Abs_600_ of these cultures. Samples of *E. coli* transformed with the empty pSB3K3 vector had approximately 1.5-fold lower Abs_600_ values than samples of cells transformed with pSB3K3_BBa_I20260. This may have resulted in relatively higher average fluorescence per cell for cultures without reporter protein compared to fluorescence per cell in cultures with GFPmut3b expressed and consequently lower difference value.

To show that the difference in optical density can explain the observed discrepancy with the pSB-derived vector, we calculated the average fluorescence per cell (F/Abs_600_) by subtracting the fluorescence of cells containing EV at the same cell density (calculated using the linear equation of the dilution series from the culture with expressed GFPmut3b, Suppl. Fig. 2a) from the fluorescence of cells expressing GFP (F(GFP) - F(EV)) and dividing the difference by Abs_600_, (F(GFP) - F(EV))/Abs_600_. With this calibration, the values for fluorescence per cell were much more comparable at different Abs_600_ values (Suppl. Fig. 2b). To avoid possible method bias, all further fluorescence measurements were performed either in cell lysates or with cell cultures diluted to the same optical density values (Abs_600_ for *E. coli* and Abs_730_ for *S. sp.* PCC 6803).


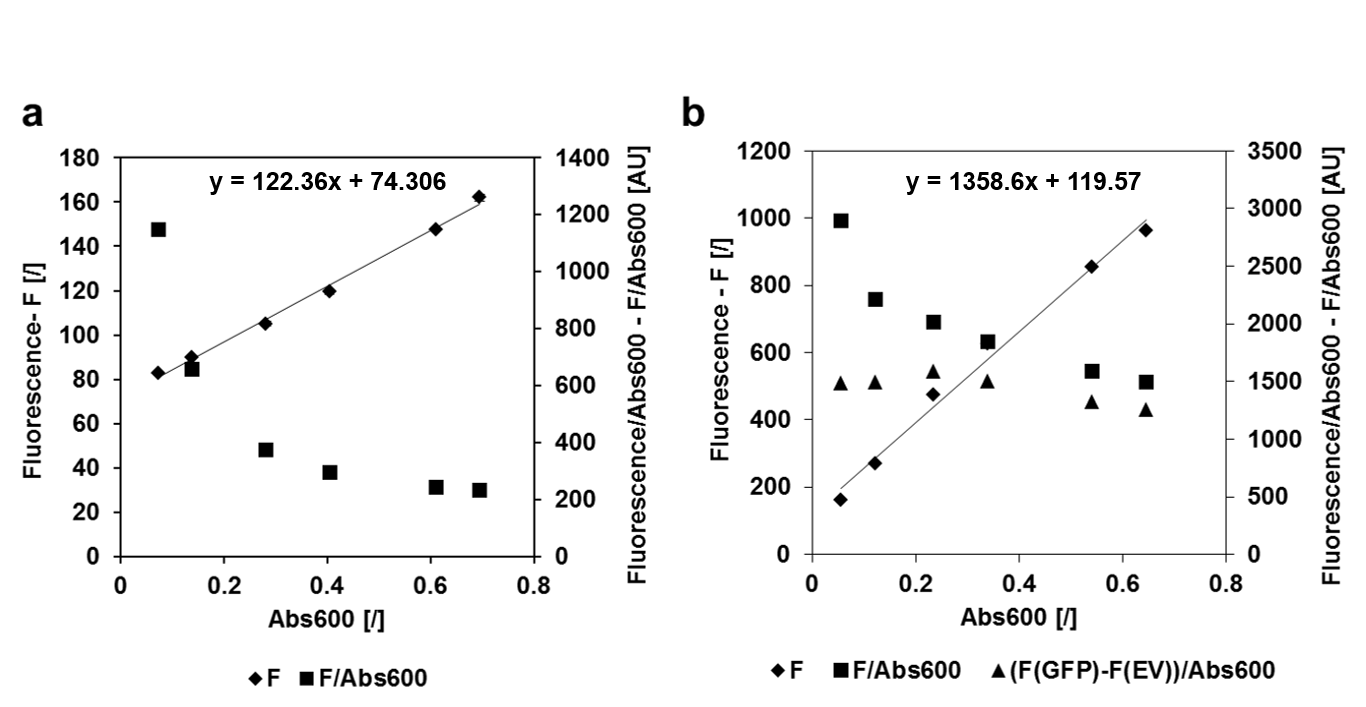


**Suppl. Art. Fig. 2 Fluorescence (F) and average fluorescence per cell values (F/Abs_600_) at different densities of *E. coli* culture. a** Fluorescence and average fluorescence per cell values for cells transformed with empty pMJc01 vector. Linear regression trend line and equation are given for fluorescence values. **b** Fluorescence and average fluorescence per cell values for cells transformed with pMJc01_L21_RBS*_*GFPmut3b.*

**GFPmut3b expressed in *E. coli* is partially insoluble and does not contribute to the measured fluorescence**

To analyse absolute GFPmut3b levels in cells harbouring different expression vectors, we performed SDS -PAGE of *E. coli* cell lysates (Suppl. Fig. 3). In most samples, GFPmut3b expression was too low to be reliably detected by Coomassie Blue staining, mainly because it overlapped with one of the intrinsic bacterial proteins. Nevertheless, in lysates of cells containing plasmids with *GFPmut3b* reporter under the control of the L21_RBS* regulatory sequence - particularly cells transformed with pSB3K3 and pMJc01 derivatives - the presence of GFPmut3b could be clearly seen.

Cell lysates were separated into soluble and insoluble fractions and analysed by SDS -PAGE, with some reporter detected in the insoluble fraction. The amount of insoluble protein was proportional to the total amount of GFPmut3b as estimated from SDS-PAGE of total cell lysates. Fluorescence measurements of the soluble and insoluble fractions showed that GFPmut3b did not fluoresce in the insoluble fraction, indicating that the fluorescence could be attributed only to the soluble protein and not to the total amount of GFPmut3b expressed.


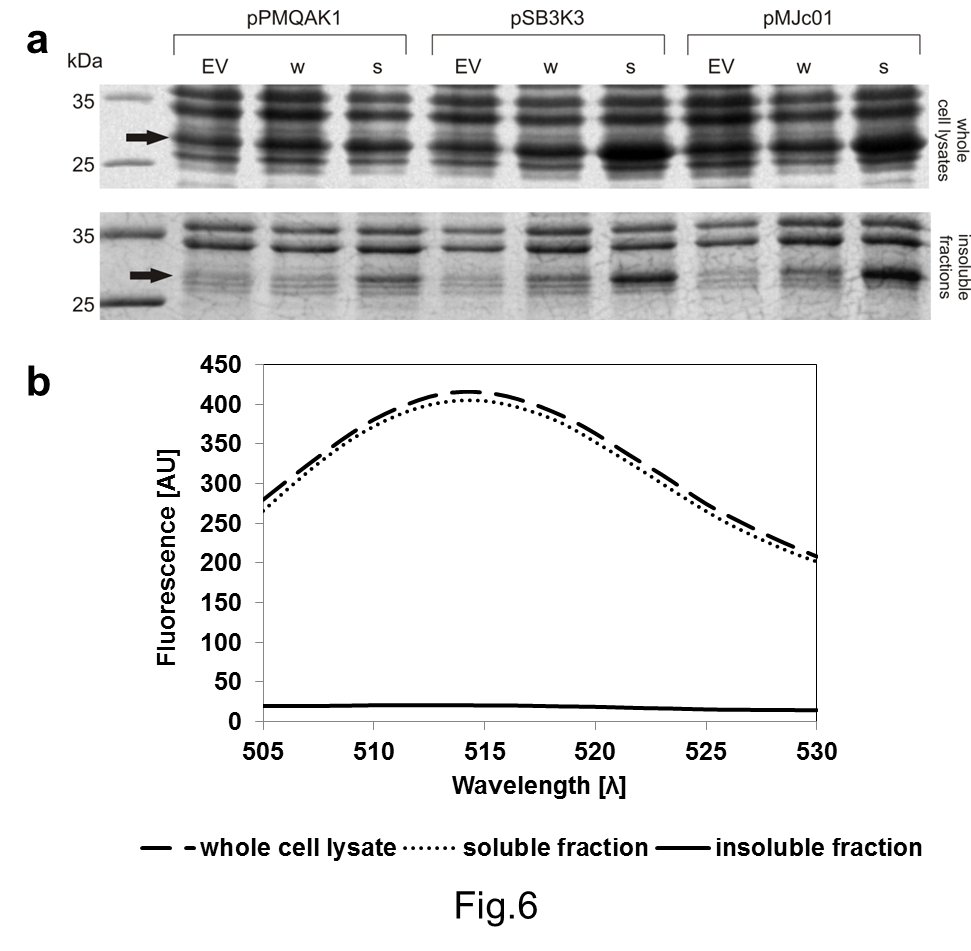


**Suppl. Art. Fig. 3 Analysis of GFPmut3b expression in *E. coli*. a** SDS-PAGE of whole cell lysates and insoluble fractions of cell lysates (w - GFPmut3b expression device with J23101_B0032 regulatory sequences, s - GFPmut3b expression device with L21_RBS* regulatory sequences). The expected size of GFPmut3b (26.9 kDa) is marked with arrows. **b** GFPmut3b fluorescence in whole cell lysates and soluble or insoluble fractions.


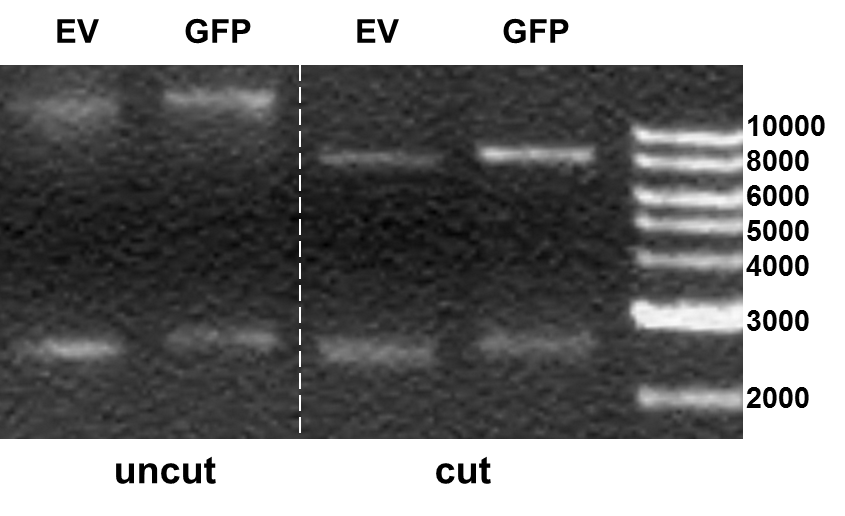


**Suppl. Art. Fig. 4 Single-stranded circular pPMQAK1 plasmid after standard isolation with alkaline lysis.** Agarose gel presenting uncut and *Eco*RI-cut pPMQAK1_EV (7689 bp) and pPMQAK1_BBa_I20260 (8615 bp) loaded after isolation from *E. coli* with an alkaline lysis-based miniprep kit. The upper band in each lane corresponds to double-stranded linear or circular pPMQAK1 and the lower band corresponds to single-stranded circular pPMQAK1.

**Optimization of *Synechocystis* sp. PCC 6803 lysis method suitable for papain inhibition assay**

Since the *Synechocystis* sp. PCC 6803 cells are more difficult to lyse compared to *E. coli*, we had to adapt our lysis protocol for preparation of cell lysates suitable for papain inhibition assay. The efficiency of different lysis methods was determined by examining the pellet and supernatant colour in centrifuged samples after applying different sonication times and using different components of the lysis buffer (namely lysozyme, DTT and Triton X-100 at final concentrations of 0.5 mg/ml, 50 mM and 1 %, respectively). Addition of DTT and Triton X‑100 in the lysis buffer inhibited papain activity and was therefore discarded. DTT also did not improve the efficiency of lysis, while Triton X-100 did, but on the other hand also presented additional hindrance as it contributed to foaming of the samples during sonication. In the end, we decided to omit also the addition of lysozyme, as it required use of a different buffering agent and pH (when working with *E. coli*, lysis buffer with 1 X TE, pH 9 was used, while in case of adding lysozyme, 100 mM phosphate buffer, pH 6.2 had to be used to ensure appropriate conditions for lysozyme activity), as well as longer processing times (additional 1 h incubation at 37 °C). After excluding the possibility of using the tested chemical additives, we focused on determining the sufficient sonication times. Used sonication time (4×1 min) was chosen as the minimum treatment that ensured complete lysis of pelleted cells from 1-2 ml of *Synechocystis* sp. PCC 6803 cell culture.
