# Supplementary figures and images for: A chimeric vector for dual use in cyanobacteria and Escherichia coli, tested with cystatin, a nonfluorescent reporter protein
Source: PeerJ. 2021 Nov 3;9:e12199. doi: 10.7717/peerj.12199 (PMC8571960; doi:10.7717/peerj.12199)

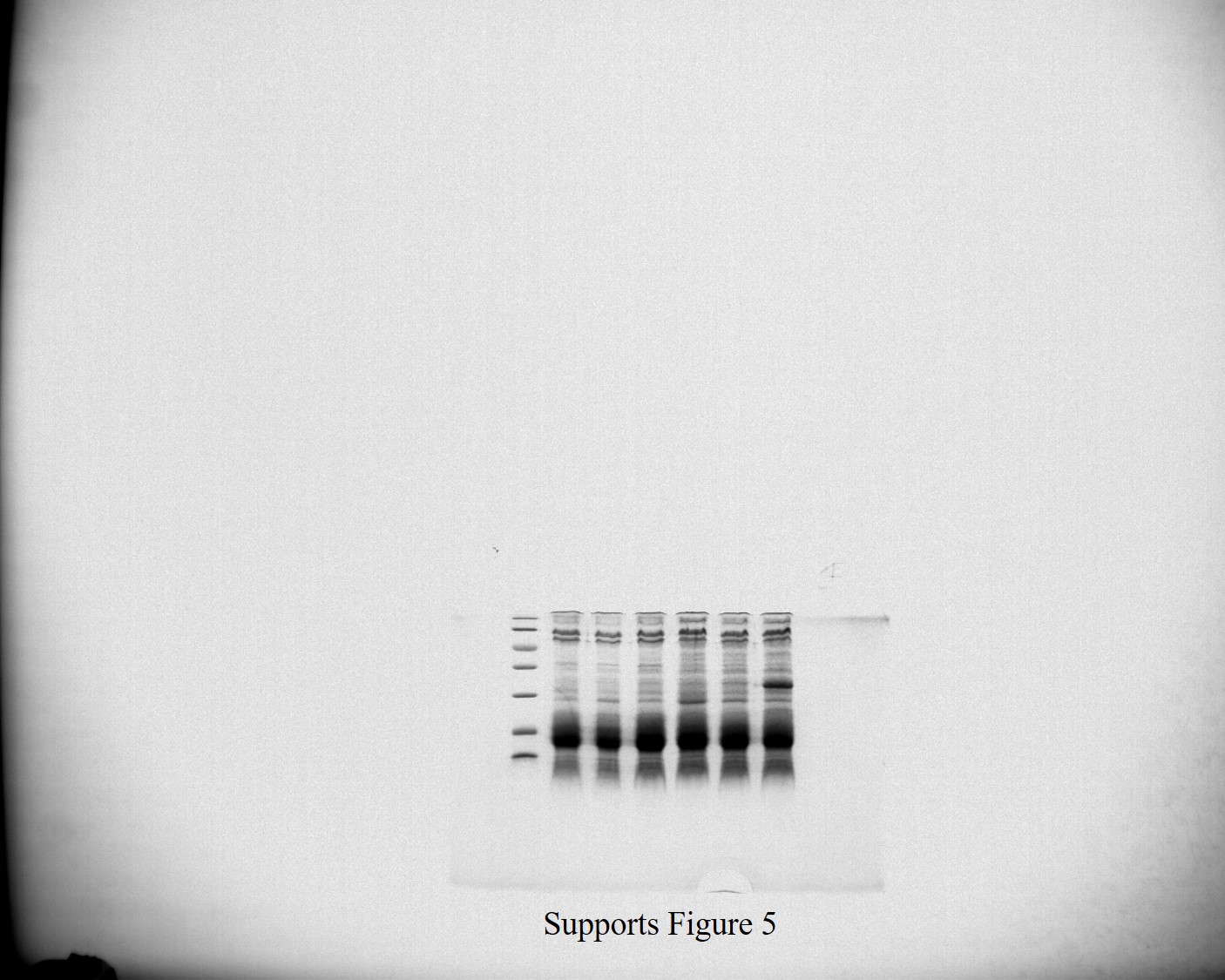

Supplement: Supplemental Information 2 [file peerj-09-12199-s002.jpg]

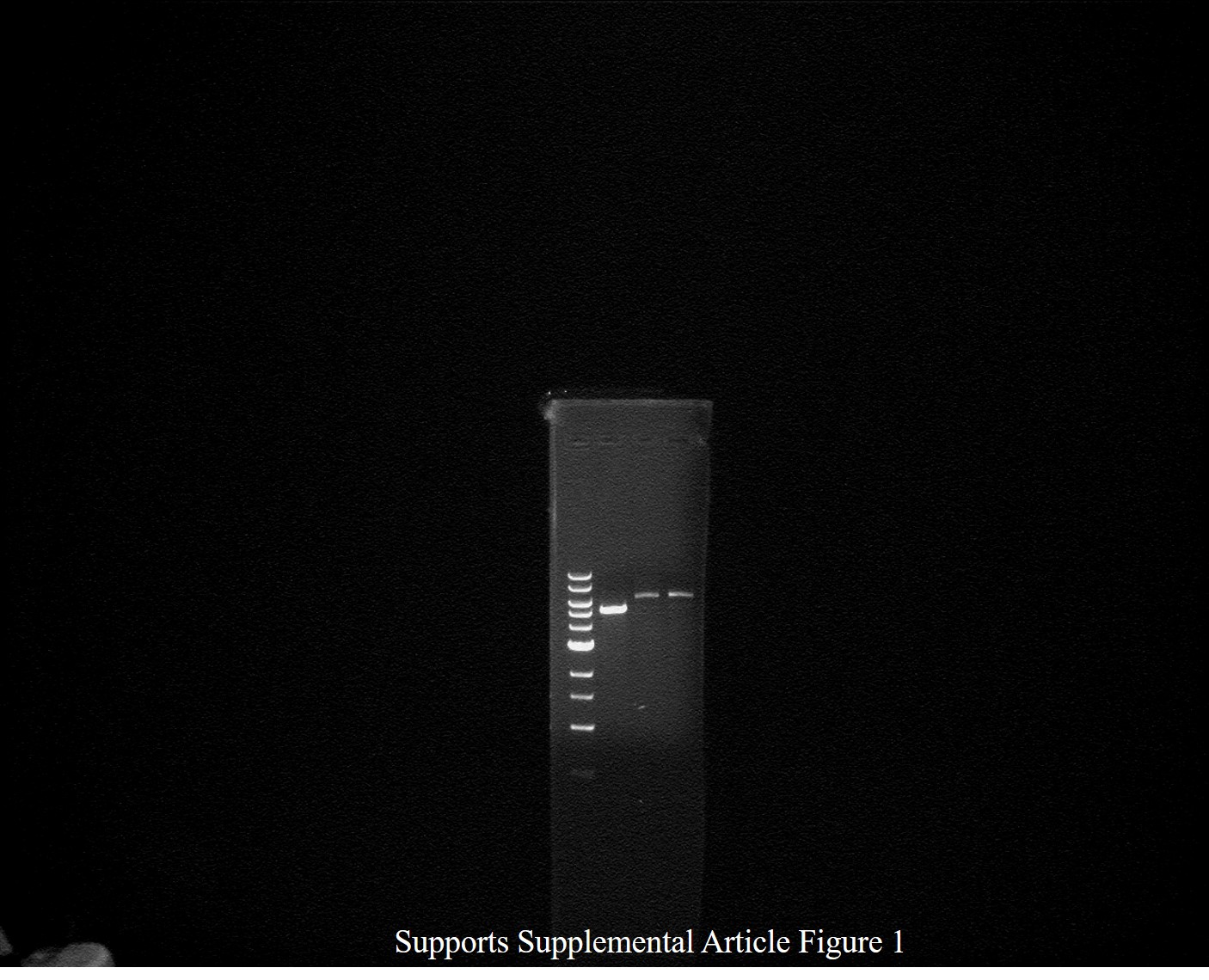

Supplement: Supplemental Information 3 [file peerj-09-12199-s003.jpg]

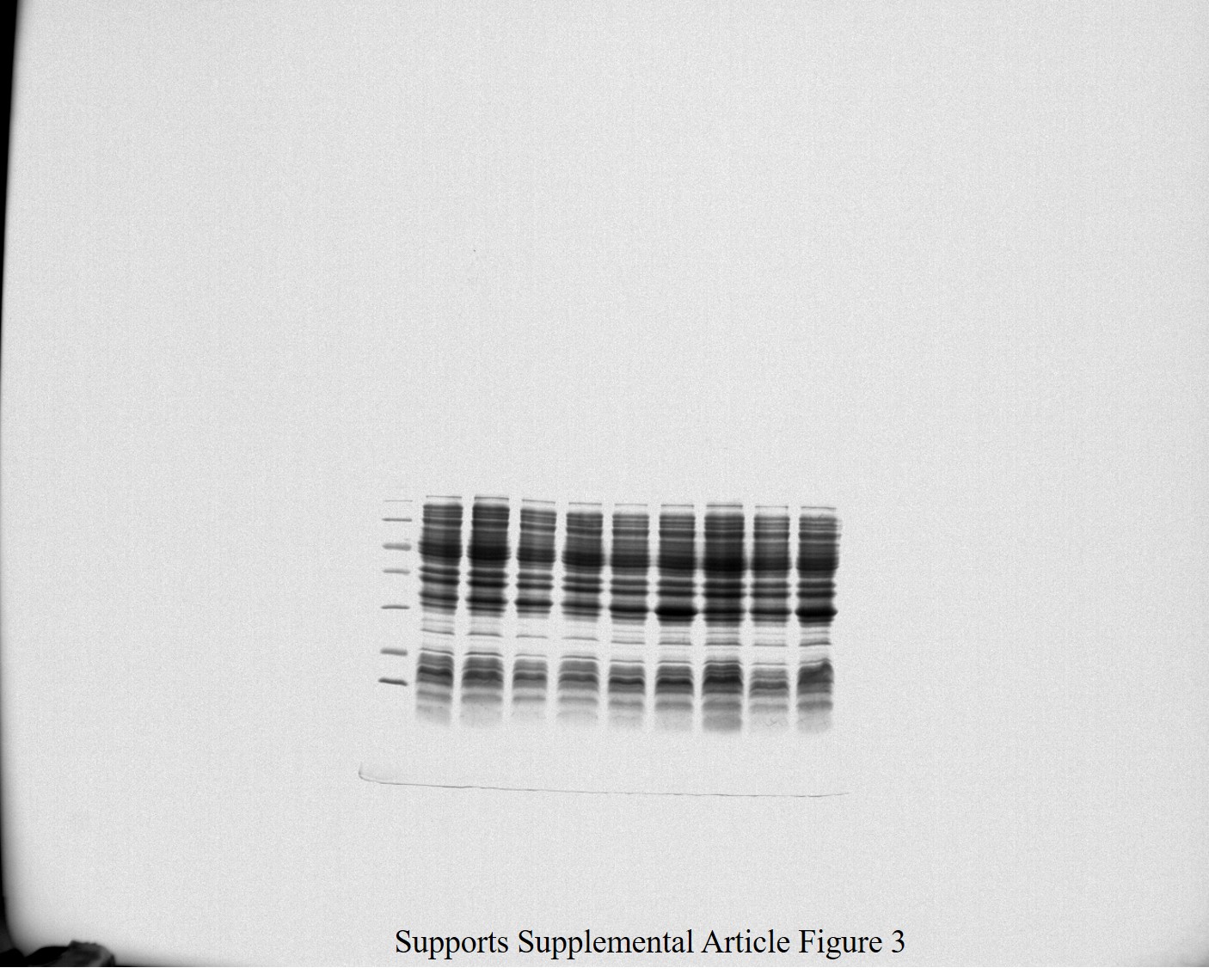

Supplement: Supplemental Information 4 [file peerj-09-12199-s004.jpg]

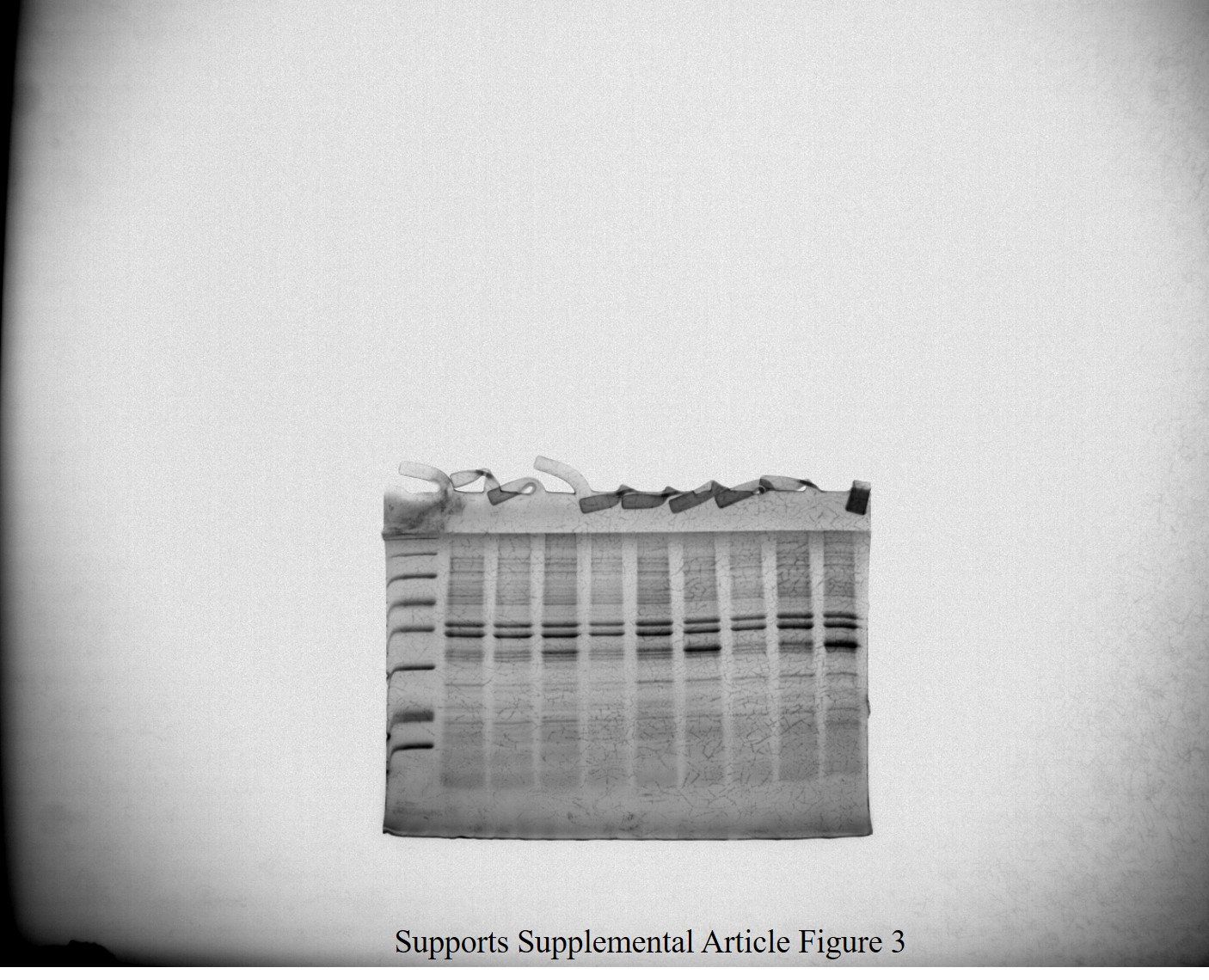

Supplement: Supplemental Information 5 [file peerj-09-12199-s005.jpg]

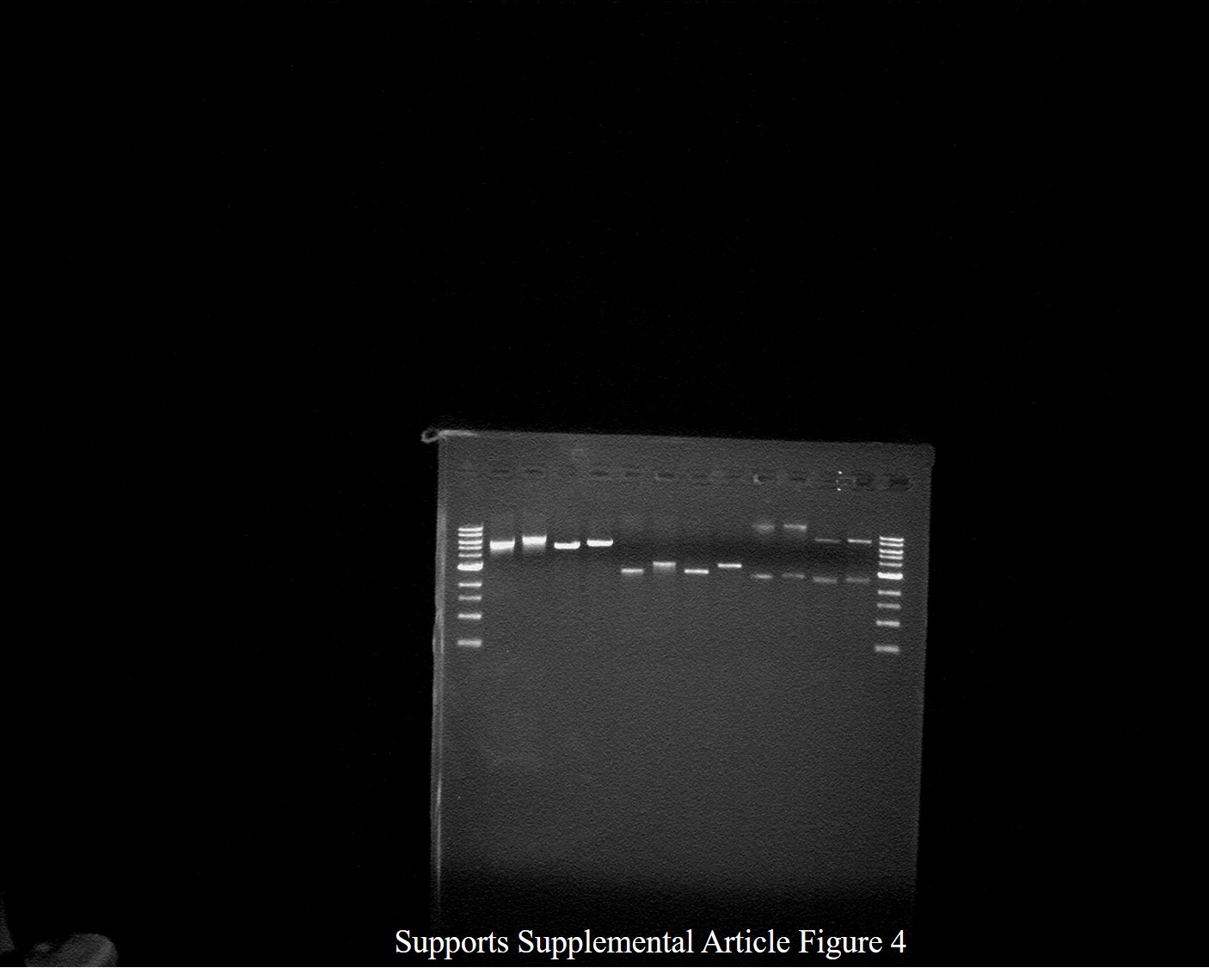

Supplement: Supplemental Information 6 [file peerj-09-12199-s006.jpg]
